# Supplementary material for: Information and communication technologies and quality of life in home confinement: Development and validation of the TICO scale
Source: PLoS One. 2020 Nov 5;15(11):e0241948. doi: 10.1371/journal.pone.0241948 (PMC7643959; doi:10.1371/journal.pone.0241948)
Supplement: S1 File — (DOC) [file pone.0241948.s001.doc]

From the **xxxxxxx** we are conducting a study on the influence of the use of Information and Communication Technologies on the Quality of Life and Personal Welfare in the current situation of confinement by the Covid-19.

It is very important for us that you answer this questionnaire; it will only take a few minutes.

THANK YOU IN ADVANCE FOR YOUR PARTICIPATION.

Your answers are very useful for the advancement of scientific knowledge in relation to situations of confinement of the population in times of crisis. It will take you a few minutes to complete the survey.

The questionnaire is completely anonymous, voluntary and disinterested. All the content of this survey is confidential and no IP addresses are recorded.

We would appreciate it if you would send the link to the questionnaire to all your contacts in order to achieve the greatest possible participation.

Consent:

I am not of legal age or do not wish to participate in the study

I am of legal age and wish to participate in the study. I have been informed that this is an anonymous, voluntary and disinterested study.

**SOCIO-DEMOGRAPHIC DATA**

**In relation to the current situation of the coronavirus pandemic, please indicate which of the following statements best reflects your current situation:**

- I am in good health and am not afraid of being infected.
- I am healthy but afraid of being infected.
- I have mild symptoms of coronavirus but have not been tested.
- I have coronavirus but no symptoms.
- I have coronavirus but they are mild symptoms.
- I have already passed the coronavirus and I am recovered.

**Gender:**

- - Female.
  - Male.

**Age:**

**……**

**Completed studies?**

- - No studies.
  - Elementary.
  - Secondary.
  - Bachelor.
  - Medium Grade FP.
  - Higher Grade FP.
  - University Studies.
  - Dr.

**Marital status.**

- - Single.
  - Married/Couple.
  - Divorced/separated.
  - Widower.

**Coexistence in the current moment of confinement*.**

- - Alone.
  - 2 persons.
  - 3 to 5 people.
  - More than 5 people.

**Employment status*.**

- - Unemployed (pre-pandemic)
  - Passive-Affected by the pandemic situation/ERTE/Self-Employed/etc.
  - Assets - Third party account.
  - Active - Self-employed.
  - Active - Civil Service.
  - Retired.
  - Student.
  - Housework.
  - Others.

**Monthly income?**

- - < 500€.
  - Between 500-1000€.
  - Between 1000-1500€.
  - Between 1500-2000€.
  - >2000€.
  - No income.
  - I prefer not to answer.

**Number of children or dependent minors:**

..........

**Number of elderly dependents:**

..........

**USE OF INFORMATION AND COMMUNICATION TECHNOLOGIES**

**Point out the technological devices you have at home during this period of confinement.**

*You can point to more than one option.*

- - - Desktop computer.
    - Laptop computer.
    - Tablet.
    - Cell phone with Internet connection (Smartphone).
    - Television without Internet connection.
    - Television with Internet connection (Smart-TV).
    - Video game consoles.

**Point out with which device(s) you connect to the Internet and social networks in the current confined situation.**

*You can indicate more than one option:*

- Computer.
- Internet or smart phone.
- Tablet.
- Television with Internet.
- Video game console.

**In the current situation of confinement, indicate the daily frequency with which you use the following means of communication and information**.

*Answer all*

*Nothing /Less than 1 hour / 1 to 2 hours / 2 to 3 hours / 3 to 4 hours/More than 5 hours*

- - - Radio.
    - Television.
    - Pay TV (Netflix, HBO, Amazon prime, etc.)
    - Newspapers/magazines in paper.
    - Press/magazines online, through the Internet.
    - Internet pages or blogs.
    - Telephone.
    - Cell phone.

**In the current situation of confinement, indicate the daily frequency with which you use the following social networks or messaging and video calling applications.**

*Nothing /Less than 1 hour / 1 to 2 hours / 2 to 3 hours / 3 to 4 hours/More than 5 hours*

- - - WhatsApp messages.
    - WhatsApp video calls.
    - WhatsApp voice calls.
    - Facebook.
    - Twitter.
    - Instagram.
    - YouTube.
    - TikTok.
    - Digital group meeting applications such as Skype/Meet google/Zoom/etc.
    - Video call game applications.

**In the current situation of confinement, what are you using the Internet, social networks and/or messaging and video calling applications for?**

*1 is "very little or nothing" and 7 "a lot".*

- - - - To see news.
      - To communicate with the family.
      - To communicate with friends.
      - To Work.
      - Playing sports.
      - See recipes/cooking tips.
      - Play (recreational activities, etc).
      - Gambling, online betting.
      - Motivate/inspire me.
      - Entertain me.
      - Watch movies/series.
      - Read.
      - Study/learning.
      - Listen to music.
      - Listen to podcast.
- Buy.

**QUALITY OF LIFE IN CONFINEMENT**

*7 points: strongly disagree (1), strongly agree (7)*

1. In most respects, ICT make my life confinement satisfactory.

2. Life in confinement has improved thanks to ITC.

3. I am more satisfied with my life in confinement when I use technologies.

4. ICT help me “important” things done in confinement.

5. If I had to live in confinement again, I would continue using ITC.

6. Thanks to ITC, I always have someone to talk to.

7. Thanks to ITC, I feel that people care about me.

8. Thanks to ITC, I can ask for help from family and friends.

9. Whenever I feel sad, I use ITC.

10. Whenever I feel lonely, I use ITC.

11. Whenever I do not fell loved, I lean on ITC.

12. When I feel bored, I turn to ITC.

13. ICT help me settle on meetings and celebrations with friends and family.

14. ICT help me have a clear purpose and direction in life.
