# Supplementary material for: Information and communication technologies and quality of life in home confinement: Development and validation of the TICO scale
Source: PLoS One. 2020 Nov 5;15(11):e0241948. doi: 10.1371/journal.pone.0241948 (PMC7643959; doi:10.1371/journal.pone.0241948)
Supplement: S2 File — (DOC) [file pone.0241948.s002.doc]

Desde XXXXX, estamos realizando un estudio sobre la **Influencia del uso de las Tecnologías de la Información y la Comunicación en la Calidad de Vida y Bienestar Personal en la actual situación de confinamiento por el Covid-19.**

Es muy importante para nosotros que contestes este cuestionario, solo te llevará unos minutos.

GRACIAS DE ANTEMANO POR TU PARTICIPACIÓN.

Tus respuestas son de gran utilidad para nuestra investigación, nos van ayudar a aumentar el conocimiento científico en relación a las situaciones de confinamiento de la población en épocas de crisis.

El cuestionario es completamente anónimo, voluntario y desinteresado. Todo el contenido de esta encuesta es confidencial y no se registra ninguna dirección IP.

Te agradeceríamos que enviaras el enlace del cuestionario a todos tus contactos para conseguir la mayor participación posible.

Consentimiento:

- No soy mayor de edad o no deseo participar en el estudio

- Soy mayor de edad y deseo participar en el estudio. He sido informado de que este es un estudio anónimo, voluntario y desinteresado.

**DATOS SOCIODEMOGRÁFICOS**

**En relación con la situación actual de pandemia por coronavirus, señale cuál de las siguientes afirmaciones refleja mejor su situación actual:**

- Me encuentro bien de salud y no tengo miedo de estar infectado.
- Me encuentro bien de salud pero tengo miedo de estar infectado.
- Tengo síntomas de coronavirus leves pero no me he hecho la prueba
- Tengo coronavirus pero no tengo síntomas
- Tengo coronavirus pero son síntomas leves
- Ya he pasado el coronavirus y estoy recuperado.

**Género:**

- Mujer
- Hombre

**Edad:**

……

**Estudios finalizados?**

- Sin estudios
- Primaria-EGB
- Secundaria-BUP
- Bachiller-COU
- FP grado medio
- FP grado superior
- Estudios universitarios
- Doctorado

**Estado civil:**

- Soltero
- Casado/pareja de hecho
- Divorciado/separado
- Viudo

**Convivencia en el momento actual de confinamiento.**

- Solo
- 2 personas
- 3 a 5 personas
- Más de 5 personas

**Estatus laboral.**

- Desempleado (previo a la pandemia)
- Pasivo-Afectados por la situación pandemia/ERTE/Autónomos/Despidos/etc
- Activo - Cuenta ajena
- Activo - Autónomo
- Activo - Funcionariado
- Jubilado
- Estudiante
- Tareas del hogar
- Otros

**Salario mensual?***

- < 500 €
- Entre 500-1000€
- Entre 1000-1500€
- Entre 1500-2000€
- >2000€
- Sin ingresos
- Prefiero no contestar

**Número de hijos o menores de edad a su cargo:**

..........

**Número de personas mayores a su cargo:***

..........

**USO DE TECNOLOGÍAS DE LA INFORMACIÓN Y COMUNICACIÓN**

**Señala los dispositivos tecnológicos que tienes en casa en este periodo de confinamiento**

Puede señalar más de una opción.

| - Ordenador de mesa |
| --- |
| - Ordenador portátil |
| - Tableta |
| - Teléfono móvil con conexión a Internet (Smartphone) |
| - Televisión sin conexión a Internet |
| - Televisión con conexión a Internet (Smart-TV) |
| - Consolas de videojuegos |

**Señale con que dispositivo/s se conecta a Internet y a las redes sociales en la actual situación de confinamiento.**

*Puede señalar más de una opción:*

- Ordenador
- Teléfono con Internet o inteligente
- Tableta
- Televisión con Internet
- Consola de videojuego

**En la situación actual de confinamiento señale la frecuencia diaria con la que utiliza los** **siguientes medios de comunicación e información.**

*Contestar todo*

*Nada /Menos de 1 hora /De 1 a 2 horas /De 2 a 3 horas / De 3 a 4 horas/Más de 5 horas*

- Radio
- Televisión
- Televisión de pago (Netflix, HBO, Amazon prime, etc)
- Periódicos/revistas en papel
- Prensa/revistas online, a través de Internet
- Páginas o blogs de Internet
- Teléfono fijo
- Teléfono móvil

**En la situación actual de confinamiento señale la frecuencia diaria con la que utiliza las siguientes redes sociales o aplicaciones de mensajería y video-llamada.**

Nada /Menos de 1 hora /De 1 a 2 horas /De 2 a 3 horas / De 3 a 4 horas/Más de 5 horas

- Whatsapp mensajes
- Whatsapp videollamadas
- Whatsapp llamadas voz
- Facebook
- Twitter
- Instagram
- YouTube
- TikTok
- Aplicaciones para reuniones digitales grupales tipo Skype/Meet google/Zoom/etc
- Aplicaciones de juegos con video-llamadas

**En la situación actual de confinamiento para qué está utilizando Internet, las redes sociales y/o aplicaciones de mensajería y video-llamada.**

*1 es “muy poco o nada” y 7 “muchísimo”*

- Para ver noticias.
- Comunicarme con la familia
- Comunicarme con los amigos
- Trabajar
- Hacer deporte
- Ver recetas/consejos de cocina
- Jugar (actividades lúdicas, etc)
- Juegos de azar, apuestas online.
- Motivarme/inspirarme
- Entretenerme
- Ver películas/series
- Leer
- Estudiar/aprender
- Escuchar música
- Escuchar podcast
- Comprar

**CALIDAD DE VIDA EN EL CONFINAMIENTO**

**Satisfacción con la vida en situación de confinamiento:**

*7 puntos: totalmente en desacuerdo (1), totalmente de acuerdo (7)*

1. En la mayoría de los aspectos, mi vida en situación de confinamiento es satisfactoria gracias al uso de las tecnologías.
2. Las circunstancias de mi vida en situación de confinamiento han mejorado con el uso de las tecnologías.
3. Estoy más satisfecho con mi vida en situación de confinamiento cuando uso las tecnologías.
4. Consigo las cosas que considero importantes, en situación de confinamiento, con la ayuda de las tecnologías.
5. Si tuviera que vivir de nuevo una situación de confinamiento seguiría usando las tecnologías.
6. En situación de confinamiento gracias a las tecnologías siempre tengo con quien comunicarme.
7. En situación de confinamiento siento que las personas se preocupan por mí, a través de las tecnologías.
8. En situación de confinamiento puedo pedir ayuda a familiares y/o amigos a través de las tecnologías.
9. En situación de confinamiento cuando me siento triste acudo a las tecnologías.
10. En situación de confinamiento cuando me siento solo recurro a las tecnologías.
11. En situación de confinamiento cuando no me siento querido me apoyo en las tecnologías.
12. En situación de confinamiento si me siento aburrido recurro a las tecnologías.
13. En situación de confinamiento quedo con mis amigos y familiares para encuentros (“quedadas”), celebraciones y fiestas a través de las tecnologías.
14. En situación de confinamiento tengo clara la dirección y el objetivo de mi vida y las tecnologías me ayudan.
