# Supplementary material for: Information and communication technologies and quality of life in home confinement: Development and validation of the TICO scale
Source: PLoS One. 2020 Nov 5;15(11):e0241948. doi: 10.1371/journal.pone.0241948 (PMC7643959; doi:10.1371/journal.pone.0241948)
Supplement: Attachment — Submitted filename: Response to Reviewers.doc [file pone.0241948.s003.doc]

**Reviewer #1: Overall**

**Method
1. Provide a justification for selecting the particular scale for adaptation.**

To measure Quality of Life, we consider three variables to be of the utmost importance in the state of confinement: loneliness and social support, psychological well-being and satisfaction with life.

Loneliness is associated with psychological and physical discomfort. During confinement many people have been alone and the only social link was through technology. We used a Scale of Solitude validated in adult population that studies family loneliness, marital loneliness and social loneliness (Cardona, Villamil, Henao, E. and Quintero, 2010), from which those items more representative of the state of loneliness and social support were adapted to the use of technologies.

We also adapted some items from the Ryff Psychological Welfare Scale, validated in Spanish populations (Díaz, Rodríguez-Carvajal, Blanco, Moreno-Jiménez, Gallardo, Valle and van Dierendonck, 2006). The adaptation of the items was taken into account according to the fundamental variables of psychological well-being: self-acceptance, positive relationships, autonomy, control of the environment, purpose in life and personal growth, all in relation to the use of technologies.

Finally, from the Life Satisfaction Scale (Diner, Emmons, Larsen and Griffin, 1985; Atienza, Balaguer and García-Merita, 2003) of the original for world population and the Spanish adaptation of the scale, items were extracted which were adapted to the situation of confinement and the use of technologies. The scale measures subjective psychological well-being, so it fits perfectly into our study for the confinement situation.

**2. More detail is needed about recruitment of participants, i.e. how were the participants targeted – through organizations? Personal contacts? Etc.
What was the survey response rate?
Was the instrument administered anonymously or did you collect identifying information?
On average, how long did it take the participants to complete the instrument?**

The process to obtain the sample was as follows: special care was taken to identify the groups, organizations and people, with whom to maintain initial contact and who could provide access to a sample that was as heterogeneous as possible according to the characteristics of the study (geographical scope Spain and all age groups from 18 years onwards). They were contacted and asked to participate in sharing and assisting in the dissemination of the survey. The web link to the survey was disseminated through

*Social networks: Twitter, Facebook and LinkedIn.

*By email.

*Through WhatsApp and Telegram groups

*An advertising campaign was carried out on Facebook, through the Business Manager platform, segmenting the population by age and geographical area.

In order to improve the response rate, invitations were sent out weekly, indicating the objective of the research project at all times, its importance, the estimated time for completion (between 5 and 6 minutes) and the contents to be answered. They were informed about the right not to participate or to leave the online form at any time, also about the confidentiality and anonymization of the data.

The response rate has not been calculated, the collection of information was stopped once the mandatory containment period ended and after reaching a reasonable participation quota.

**3. Conclusions
Given that the results indicated that age and gender were significant factors, comment on the differences in the conclusions.**

Regardless of gender or age, the use of technologies during confinement has a positive influence on the assessment of quality of life. However, when we talk about emotions such as sadness, loneliness or the need to feel "loved", the use of technologies as a resource, support or personal relief shows a wide range of opinions that respond to age and gender differences.

**4. Include recommendations for future research in this area.**

Future lines of research:

*Influence of ICTs on the modification of behaviour and motivational levels

*Perception of the time of use of ICTs and real use of them, differentiating the different social networks by use/consumption, taking into account age and gender

*Study of the perception of Quality of Life in the post-Covid-19 /post confinement era.
